# Dynamic Changes of Ovarian Masses During Pregnancy and the Effects on Pregnancy Outcomes

**DOI:** 10.1155/nrp/6401831

**Published:** 2026-06-01

**Authors:** Shengjie Zhou, Shuangjia Pan, Xiaosheng Li, Zhuya Ying, Rong Ma, Yuqing Jin, Xueqiong Zhu, Xuejie Zhu

**Affiliations:** ^1^ Department of Obstetrics and Gynecology, Zhejiang Provincial Clinical Research Center for Obstetrics and Gynecology, The Second Affiliated Hospital of Wenzhou Medical University, Wenzhou, 325027, Zhejiang, China, wmu.edu.cn; ^2^ Department of Obstetrics and Gynecology, Taizhou Maternal and Child Health Hospital, Taizhou, 318000, Zhejiang, China; ^3^ Department of Obstetrics and Gynecology, The First Affiliated Hospital of Wenzhou Medical University, Wenzhou, 325015, Zhejiang, China, wzhospital.cn

**Keywords:** dynamic changes, management methods, ovarian masses, pregnancy outcomes

## Abstract

**Background:**

To investigate the dynamic changes in ovarian mass size during pregnancy and the impact of different management methods for ovarian masses on pregnancy outcomes.

**Methods:**

A multicenter retrospective analysis of 177 pregnant women with ovarian masses (142 follow‐up and 35 surgical cases) assessed mass size via ultrasound, analyzed its dynamic changes and risk factors, reviewed management impacts on perinatal outcomes, and calculated pathological type distributions.

**Results:**

Compared to first trimester, the maximum diameter and volume of ovarian masses significantly decreased in the third trimester. Furthermore, compared to third trimester, the maximum diameter and volume of these masses were further reduced postpartum (*p* < 0.05). Multivariate regression demonstrated that age (OR [95% CI]: 0.888 [0.788–0.999]) and prepregnancy BMI (OR [95% CI]: 1.179 [1.013–1.373]) were factors influencing the increase in the size of ovarian masses during pregnancy. Pregnancy outcomes in the surgery‐during‐pregnancy group did not find significant difference compared to the follow‐up group (*p* > 0.05). Additionally, the predominant pathological type of ovarian mass during pregnancy was mature cystic teratoma (40%), and the overall malignancy rate of the ovarian masses was 2.5%.

**Conclusions:**

The size of ovarian masses showed a decreasing trend as pregnancy progresses. Higher prepregnancy BMI and younger age were risk factors for the increase in ovarian mass size during pregnancy. Furthermore, when performed appropriately, surgical intervention for ovarian masses during pregnancy was safe. Mature cystic teratoma was the most prevalent pathological entity of ovarian mass treated surgically during pregnancy.

## 1. Introduction

With the rising employment of ultrasound in the first trimester, the detection rate of ovarian masses in pregnant women has gradually increased [1]. Studies have shown that the incidence of ovarian masses during pregnancy ranged from 0.2% to 2%, which was approximately 2 to 20‐fold higher than that in the general age‐matched women [2].

Ovarian masses during pregnancy are primarily classified into physiological masses (such as functional cysts and corpus luteum cyst) and pathological masses (such as mature cystic teratoma and ovarian cancer) [3]. Approximately 70%–80% of ovarian masses during pregnancy were physiological cysts [4, 5]. Additionally, the malignancy rate of ovarian masses in pregnant women was 2%–6% [6, 7]. Although most ovarian masses were benign, the incidence of pedicle torsion in pregnant women with ovarian masses was high [8, 9]. Given the varied types of ovarian masses during pregnancy, it is essential to understand dynamic changes of masses to guide appropriate clinical management.

Therapeutic approaches for ovarian masses during pregnancy encompass both surgical intervention and conservative management. Larger ovarian masses might compress the pregnant uterus, leading to fetal growth restriction, preterm birth, or fetal abnormalities [5, 10]. Thus, some studies recommended exploratory surgery for symptomatic ovarian masses or ovarian masses exceeding 6 cm in the second trimester [1, 11]. Furthermore, surgery could relieve mass compression, rule out malignancies, and prevent pregnant complications such as ovarian mass rupture, torsion, and labor obstruction [12]. Moreover, exclusive reliance on conservative management could result in irreversible damage to function of ovarian tissue [13]. Therefore, timely and appropriate management is crucial for pregnant women with ovarian masses.

This study aims to explore the dynamic changes of ovarian mass size in pregnant women and to observe impact of different treatment methods to ovarian masses on pregnancy outcomes, which enhance the accuracy of screening and diagnostic methods for ovarian masses during pregnancy and support the development of effective clinical treatments for ovarian masses.

## 2. Methods

A multicenter retrospective study enrolled pregnant women who had at least one ovarian mass detected by ultrasound in early pregnancy at the participating hospitals from January 2018 to October 2023. Pregnant women were excluded if they had incomplete clinical data, multiple pregnancies, nonovarian origin of the mass, multiple ovarian masses on the same side, or maternal comorbidities such as chronic hypertension, chronic diabetes, and other major organ diseases. Based on the management of ovarian masses during pregnancy, the enrolled pregnant women were classified into the surgery‐during‐pregnancy group and the follow‐up group.

The characteristics of the enrolled pregnant women were recorded, including age, prepregnancy body mass index (BMI), parity, gravidity, conception method, and history of ovarian masses. Pregnancy outcomes were recorded, including the occurrence of hypertensive disorders of pregnancy (HDP, including gestational hypertension defined as systolic BP ≥ 140 mmHg and/or diastolic BP ≥ 90 mmHg after 20 weeks of pregnancy) and preeclampsia (characterized by hypertension, proteinuria, and/or end‐organ damage; this group also encompasses women with associated conditions such as HELLP syndrome), gestational diabetes mellitus (GDM, defined as fasting blood glucose ≥ 5.1 mmol/L, or1‐hour postrandial plasma glucose ≥ 10.0 mmol/L, or 2‐hour postrandial plasma glucose ≥ 8.5 mmol/L at 24–28 weeks of gestation), premature rupture of membranes (PROM, defined as rupture of membranes prior to the onset of labor), pedicle torsion (defined as the anatomical displacement of the ovary or fallopian tube along the axis of the infundibulopelvic ligament and the ovarian ligament during pregnancy), as well as delivery method, gestational age at delivery, neonatal Apgar scores, neonatal birth weight, and postoperative pathology of ovarian masses.

**FIGURE 1:**
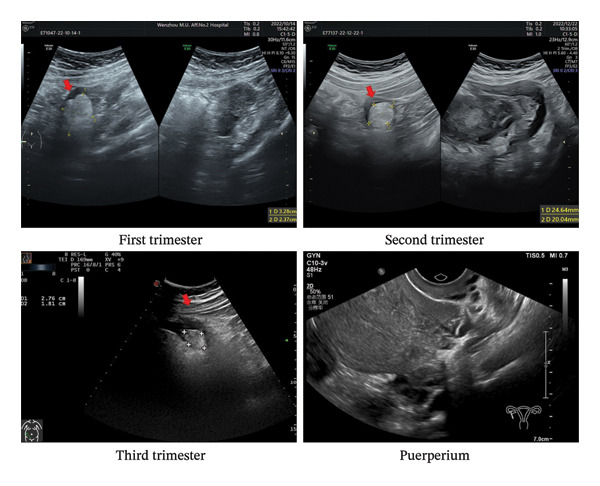
Ultrasound images of a single pregnant woman’s ovarian mass at first trimester, second trimester, third trimester, and puerperium. *Arrows* indicated the location of ovarian mass.

Additionally, ultrasound data on ovarian masses were collected, including the lengths along three axes (a, b, c) in the first trimester (the first 14 weeks), second trimester (14–27^+6^ weeks), third trimester (≥ 28 weeks), and puerperium (Figure 1). The maximum diameter was defined as the largest value among the lengths a, b, and c. The change in maximum diameter (ΔD) was defined as the difference between the maximum diameter at the latter ultrasound (LD) and the former maximum diameter (FD) (ΔD = LD‐FD). Considering the measurement errors associated with ultrasound, a ΔD greater than 1 cm or less than −1 cm was defined as an increase or decrease in maximum diameter, respectively, while changes between −1 cm and 1 cm were considered no change [14]. The ellipsoid formula (volume (cm^3^) = (4π)/3 × *a*/2 × *b*/2 × *c*/2 =  (*a* *·* *b* *·* *c*·π)/6) was used to calculated the volume of the ovarian masses. The volume change (ΔV) was defined as the difference between the volume at the latter ultrasound (LV) and the former volume (FV) (ΔV = LV − FV). The growth rate (GR) of volume was calculated as GR = ΔV/FV × 100% [15]. Considering the measurement errors associated with ultrasound, a GR greater than 20% was defined as a volume increase, a decrease exceeding 20% was defined as a volume decrease, and a volume change within 20% was defined as no significant change [14].

All ultrasound examinations were assessed by senior sonographers with more than 10 years of dedicated experience in gynecological ultrasound, who are specialists in gynecologic imaging. All pathological specimens were evaluated by dedicated pathologists with over 15 years of subspecialty expertise in gynecologic pathology, affiliated with the Department of Pathology at participating hospitals. They have extensive expertise in diagnosing ovarian tumors, ensuring the reliability of histopathological evaluation. Histopathological diagnosis was regarded as the gold standard for final classification in this study.

Data were analyzed using SPSS Version 26.0. Levene’s test and the Shapiro–Wilk normality test were performed to assess homogeneity of variances and normality. Continuous variables that met normal distribution and homogeneity of variance were expressed as mean ± standard deviation (SD) and analyzed by the independent samples *t*‐test. Data that did not meet these criteria were expressed as median (interquartile range, IQR) and analyzed using the Mann–Whitney *U* test. The chi‐square test or Fisher exact test was used to compare categorical variables. With results presented as odds ratios (ORs) and 95% confidence intervals (CIs), logistic regression was used to analyze the factors influencing the volume changes of ovarian masses during pregnancy. Statistical significance was set at *p* < 0.05.

## 3. Results

A total of 177 pregnant women with ovarian masses were included in the study, accounting for 189 ovarian masses. Among them, 142 pregnant women with 149 ovarian masses were in the follow‐up group, while 35 pregnant women with 40 ovarian masses were in the surgery‐during‐pregnancy group. The mean age of all included pregnant women was 29.70 ± 4.25 years, with an average prepregnancy BMI of 21.03 ± 2.57 kg/m^2^. The mean birth weight of newborns was 3329.04 ± 429.38 g, and the median gestational age at delivery was 39 weeks, with a quartile range of 38–40 weeks.

### 3.1. Changes in Ovarian Mass Size During Pregnancy

Changes in the size of ovarian masses during different trimesters in the follow‐up group were evaluated by examining the maximum diameter and volume (Table S1). Compared to first trimester, 59.06% (88/149) of ovarian masses showed no change in maximum diameter, 10.07% (15/149) increased, 4.70% (7/149) decreased, and 26.17% (39/149) disappeared in the second trimester. In comparison to second trimesters, 51.68% (77/149) of ovarian masses had no change in maximum diameter, 8.05% (12/149) increased, 7.38% (11/149) decreased, and 32.89% (49/149) disappeared in the third trimester. From first to third trimesters, 22.15% (33/149) of ovarian masses remained the same in maximum diameter, 27.52% (41/149) increased, 17.45% (26/149) decreased, and 32.88% (49/149) disappeared. In addition, 18.69% (20/107) of ovarian masses had no change in maximum diameter, 4.67% (5/107) increased, 20.56% (22/107) decreased, and 56.08% (60/107) disappeared from third trimester to puerperium. Additionally, comparing the maximum diameter of ovarian masses at different trimesters, the results indicated a significant decrease from first to second trimester, from first to third trimester, and from third trimester to puerperium (*p* < 0.05) (Table 1).

**TABLE 1:** Maximum diameter and volume changes of ovarian masses during pregnancy.

	ΔD of maximum diameter (mm)	p*^a^*	ΔV of mass volume (cm^3^)	p*^b^*
First to second trimesters	−1.00 (−22.00, 4.00)	< 0.001	−1.81 (−6.68, 3.71)	0.068
Second to third trimesters	0.00 (−4.00, 3.00)	0.563	0.00 (−5.85, 1.96)	0.184
First to third trimesters	−4.00 (−26.00, 3.00)	< 0.001	−2.74 (−10.88, 2.17)	0.002
Third trimester to puerperium	0.00 (−7.00, 0.00)	< 0.001	0.00 (−3.18, 0.00)	0.016

*Note:* Data were presented as median (IQR).

^a^Comparison of maximum diameter changes in ovarian masses during pregnancy.

^b^Comparison of volume changes in ovarian masses during pregnancy.

Analyzing the change of ovarian mass volume, 53.69% (80/149) of ovarian masses had no change in volume, 13.42% (20/149) increased, 6.71% (10/149) decreased, and 26.18% (39/149) disappeared from first to second trimesters. From second to third trimesters, 16.11% (24/149) of ovarian masses showed no change in volume, 24.83% (37/149) increased, 26.17% (39/149) decreased, and 32.89% (49/149) disappeared. Compared to first trimester, 18.12% (27/149) of ovarian masses had no change in volume, 26.17% (39/149) increased, 22.82% (34/149) decreased, and 32.89% (49/149) disappeared in the third trimester. Compared to third trimester, 17.76% (19/107) of ovarian masses in puerperium did not alter in volume, 10.28% (11/107) increased, 14.95% (16/107) decreased, and 57.01% (61/107) disappeared. Furthermore, the results showed that the mass volume from first to third trimesters and from third trimester to puerperium was significantly reduced when comparing the volume among different trimesters (*p* < 0.05) (Table 1).

### 3.2. Risk Factors for Increase in the Volume of Ovarian Masses During Pregnancy

As shown in Table 2, multivariate logistic regression analysis indicated that age, parity, gravidity, prepregnancy BMI, history of ovarian tumor, and conception method do not affect the volume changes of ovarian masses from first to second trimesters, from second to third trimesters, or from third trimester to puerperium (*p* > 0.05). However, from first to third trimesters, a younger age (OR [95% CI]: 0.888 [0.788–0.999], *p* = 0.049) and a higher prepregnancy BMI (OR [95% CI]: 1.179 [1.013–1.373], *p* = 0.033) were associated with a higher incidence of the increase in ovarian mass volume. Across the entire pregnancy from early to late gestation, each 1 year increase in age was associated with an 11.2% decreased risk of > 20% ovarian mass volume growth (0.888 [0.788–0.999], *p* = 0.049), while each 1 kg/m^2^ increase in prepregnancy BMI was associated with a 17.9% increased risk (1.179 [1.013–1.373], *p* = 0.033).

**TABLE 2:** Logistic regression analysis of variables associated with increase in the volume of ovarian masses^a^.

	First to second trimesters OR (95% CI)	p*^a^*	Second to third trimesters OR (95% CI)	p*^a^*	First to third trimesters OR (95% CI)	p*^a^*	Third trimester to puerperium OR (95% CI)	p*^a^*
Age (year)	0.946 (0.846–1.054)	0.315	0.963 (0.859–1.080)	0.519	0.888 (0.788–0.999)	0.049	0.950 (0.786–1.163)	0.956
Prepregnancy BMI (kg/m^2^)	1.097 (0.953–1.264)	0.198	1.012 (0.865–1.185)	0.881	1.179 (1.013–1.373)	0.033	0.870 (0.625–1.219)	0.873
Gravidity	0.920 (0.580–1.459)	0.722	0.645 (0.365–1.142)	0.132	0.794 (0.467–1.350)	0.394	0.818 (0.353–1.893)	0.818
Parity	1.060 (0.402–2.795)	0.906	2.601 (0.869–7.781)	0.087	1.943 (0.674–5.599)	0.219	2.433 (0.455–13.012)	2.433
History of ovarian tumor	1.032 (0.422–2.525)	0.945	1.202 (0.435–3.322)	0.722	1.550 (0.550–4.374)	0.407	0.533 (0.117–2.435)	0.533
Conception method	0.390 (0.127–1.195)	0.099	2.083 (0.428–10.14)	0.364	0.746 (0.205–2.712)	0.656	0.846 (0.072–9.912)	0.846

*Note:* Data were presented as OR (95% CI). In logistic regression, the GR of volume increase greater than 20% between two different pregnancy stages was assigned a value of 1; otherwise, it was assigned 0.

Abbreviations: BMI, body mass index; CI, confidence interval; OR, odds ratio.

^a^Increase in the volume was defined as a growth rate (GR) of ovarian masses during pregnancy greater than 20%.

### 3.3. Association Between Diameter of Ovarian Masses and the Occurrence of Pedicle Torsion

All enrolled ovarian masses were analyzed to investigate the relationship between the diameter of ovarian masses during pregnancy and the occurrence of ovarian torsion. Ovarian masses were categorized into two groups based on their maximum diameter, defined as either the maximum diameter of the ovarian mass recorded on the most recent ultrasound prior to pedicle torsion or the maximum diameter observed by ultrasound at any point during pregnancy: one group with maximum diameters ≥ 6 cm and the other with maximum diameters < 6 cm. The results indicated that pregnant women with ovarian masses ≥ 6 cm in maximum diameter had a higher incidence of ovarian torsion compared to those with masses < 6 cm (*p* < 0.05) (Table 3).

**TABLE 3:** Relationship between ovarian mass diameter and incidence of pedicle torsion.

	Diameter of mass ≥ 6 cm (*n* = 60)	Diameter of mass < 6 cm (*n* = 129)	*p*
With pedicle torsion, *n* (%)	7 (11.7%)	2 (1.6%)	0.004
Without pedicle torsion, *n* (%)	53 (88.3%)	127 (98.4%)	

*Note:* Data were presented as *n* (%).

### 3.4. Comparison of Influencing Factors and Pregnancy Outcomes Between the Follow‐Up and Surgery‐During‐Pregnancy Groups

As shown in Table 4, no significant differences were found in the general characteristics of pregnant women with ovarian masses between the surgery‐during‐pregnancy group and the follow‐up group (including age, prepregnancy BMI, gravidity, and parity) (*p* > 0.05). However, the cesarean section rate was significantly higher in the surgery‐during‐pregnancy group compared to the follow‐up group (*p* < 0.05). Furthermore, no significant differences were found in incidence of HDP, PROM, GDM, gestational age at delivery, 5‐min Apgar score, and birth weight of neonates between the surgery‐during‐pregnancy group and the follow‐up group (*p* > 0.05).

**TABLE 4:** Comparison of influencing factors and pregnancy outcomes of different treatment modalities for ovarian masses during pregnancy.

	Follow‐up group, *n* = 142 (149)^a^	Surgery‐during‐pregnancy group, *n* = 35 (40)^a^	*p*
*Mothers*			
Age (year)	29.90 ± 4.083	28.89 ± 4.855	0.206
Prepregnancy BMI (kg/m^2^)	21.12 ± 2.51	20.64 ± 2.73	0.327
Gravidity	2 (1, 2)	1 (1, 3)	0.104
Parity	0 (0, 1)	0 (0, 1)	0.202
Cesarean section, *n* (%)	40 (28.17)	17 (48.57)	0.021
HDP, *n* (%)	9 (6.04)	0 (0.00)	0.272
PROM, *n* (%)	26 (17.45)	9 (25.71)	0.325
GDM, *n* (%)	15 (10.07)	2 (5.71)	0.383

*Neonates*			
Gestational age at delivery (weeks)	39 (38, 40)	39 (38, 40)	0.972
5‐min Apgar score	10 (10, 10)	10 (10, 10)	0.261
Birth weight (g)	3333.52 ± 430.22	3310.86 ± 431.71	0.781

*Note:* Data were presented as mean ± SD, median (IQR), or *n* (%).

Abbreviations: BMI, body mass index; GDM, gestational diabetes mellitus; HDP, hypertensive disorders of pregnancy; PROM, premature rupture of membranes.

^a^ *n* = number of pregnant women (number of ovarian masses).

### 3.5. Pathological Types of Ovarian Masses During Pregnancy

Postoperative pathological results of 40 ovarian masses in the surgery‐during‐pregnancy group were summarized. There were 6 physiological cysts (6/40, 15.0%) and 34 pathological cysts (34/40, 85.0%). Among the pathological cysts, 32 were benign (32/40, 80.0%), 1 was borderline tumor (1/40, 2.5%), and 1 was malignant (1/40, 2.5%). The only borderline tumor was a right‐sided serous borderline ovarian tumor (SBOT), initially treated with right ovarian cystectomy at 13 weeks of gestation. At the time of cesarean section, a left ovarian cyst was identified, and the patient subsequently underwent left ovarian cystectomy along with right ovarian biopsy. Final histopathology confirmed a benign mucinous cystadenoma in the left ovary and fibrovascular tissue in the right ovary, with no evidence of stromal invasion or malignant transformation in either ovary. One case of malignant transformation was identified, with a pathological diagnosis of serous ovarian cancer. In addition, the most common type of the pathological masses was mature cystic teratoma, accounting for 16 cases (16/40, 40.0%) (Table S2).

## 4. Discussion

This study indicated a decreasing trend in the maximum diameter and volume of ovarian masses during pregnancy. Multivariate regression analysis showed that younger age and higher prepregnancy BMI were independent risk factors of increased ovarian mass volume during pregnancy. The incidence of ovarian torsion was significantly associated with the size of the ovarian masses during pregnancy. The impact on pregnancy outcomes was similar between follow‐up and surgery management of pregnant women of ovarian masses. Additionally, pathological results from the surgery‐during‐pregnancy group indicated that mature cystic teratoma was the most common pathological type of ovarian masses.

This study observed the size of ovarian masses in different gestational periods (first trimester, second trimester, third trimester, and puerperium) and compared the dynamic changes in the size of ovarian masses throughout the entire pregnancy. The results showed that the size of ovarian masses during pregnancy gradually decreases from first trimester to third trimester and from third trimester to puerperium. Although previous studies have indicated that the majority of physiological ovarian masses regress during pregnancy [5, 16], which was similar with our results, no research has previously reported the dynamic changes in the size of ovarian masses during different stages of pregnancy. Pregnant patients with ovarian masses > 6 cm in diameter should be referred to specialized centers with expertise in ovarian mass sonography, given their higher risk of malignant ovarian neoplasms [17]. Therefore, this study provided the first evidence on the dynamic changes of ovarian masses throughout pregnancy.

The levels of human chorionic gonadotropin (hCG) in pregnant women rose exponentially, peaking at around 10 weeks of gestation, then declining to a plateau until delivery. The hCG continuously stimulated the corpus luteum to secrete large amounts of progesterone. This sustained stimulation could lead to the formation of corpus luteum cysts, the common type of ovarian mass during pregnancy [18, 19]. As pregnancy progresses and hCG level fluctuates, corpus luteum cysts regressed accordingly, which explained the gradual regression of ovarian masses during pregnancy.

Despite the overall trend of decreasing ovarian mass size during pregnancy, a minority of pregnant women in this study exhibited a gradual increase in the volume of their ovarian masses. The median GR of ovarian mass volume between second trimester and third trimester was 0.00% (−36.47%, 19.83%), indicating that volume of some ovarian masses in pregnant women still showed a tendency to increase. Our results indicated that higher prepregnancy BMI and younger age were independent risk factors for increased volume of ovarian masses during pregnancy. There was a correlation between higher BMI and abundant adipose tissue and chronic inflammatory response [20]. Adipose tissue, as an important endocrine organ, secretes various hormones and cytokines such as estrogen and inflammatory factors [20]. Abnormal hormone secretion may be a potential mechanism promoting ovarian masses. Inflammatory factors could affect ovarian endocrine function [21], further promoting the enlargement of ovarian masses. Additionally, it has been reported that the size of ovarian masses in nonpregnant women was associated with younger age [22]. Similarly, our study found the same correlation in cases of ovarian masses during pregnancy. The potential mechanism might be that younger women have higher levels of estrogen and progesterone and better ovarian function, which stimulate the proliferation of ovarian cells and the growth of ovarian masses, with the ovaries being more sensitive to hormonal stimulation [23].

Pedicle torsion of ovarian mass was an acute condition defined as the rotation of the ovarian pedicle around its axis, potentially leading to ovarian ischemia and eventually necrosis [7]. Studies reported that the incidence of pedicle torsion in ovarian masses during pregnancy was approximately 15% as the adnexa gradually shift out of the pelvis [24, 25]. Pedicle torsion was more common in the first trimester and the puerperium, possibly due to the rapid enlargement of the uterus in the first trimester and its rapid shrinkage during puerperium [26]. Other studies had shown similar results, indicating that the rate of pedicle torsion was higher for ovarian masses between 6 and 8 cm in diameter [25]. Our results indicated that the incidence of pedicle torsion was closely related to the size of the ovarian masses during pregnancy, with masses larger than 6 cm being more prone to pedicle torsion. Therefore, it is crucial to closely monitor ovarian masses larger than 6 cm during first trimester and the puerperium to prevent pedicle torsion.

Studies suggested that expectant management was recommended for most pregnant women with asymptomatic ovarian cystic masses. Additionally, a minority of cases required surgical intervention due to severe symptoms, torsion risk, or suspected malignancy [13, 27]. However, due to the higher risk of miscarriage following surgery for ovarian masses in early pregnancy, it is recommended to undergo surgery during the second trimester if there were no acute complications [10, 28]. Furthermore, laparoscopic surgery was more recommended for ovarian masses during pregnancy in the second trimester, as evidence indicated that laparoscopic surgery was related to a lower risk of postoperative complications in pregnant women with ovarian masses compared to open surgery [29].

Lee et al. [30] found that the risk of preterm birth was significantly higher in pregnant women with ovarian masses who underwent emergency surgery compared to those managed conservatively. Nevertheless, other studies indicated that elective surgery during pregnancy was safe and does not increase the incidence of miscarriage, PROM, or preterm birth [13, 27], and our study corroborated these findings. All pregnant women enrolled in our study were divided into follow‐up and surgery‐during‐pregnancy groups, and the finding showed that no significant differences were observed in pregnancy outcomes between the groups. The cesarean section rate was lower in the follow‐up group than in the surgery‐during‐pregnancy group. Furthermore, all patients in the follow‐up group who underwent cesarean section had concurrent mass removal, whereas only one patient in the surgery‐during‐pregnancy group required repeat mass removal at the time of cesarean delivery. This finding suggests that the higher cesarean section rate observed in the surgery‐during‐pregnancy group is more likely related to underlying clinical characteristics and obstetric decision‐making rather than the presence of the adnexal mass itself or the need for its removal. Therefore, surgical and conservative management showed no significant differences in gestational age at delivery, neonatal 5‐min Apgar score, and birth weight in pregnant women with ovarian masses, and individualized treatment should be chosen based on the specific circumstances.

Majority of ovarian masses detected during pregnancy were benign. The most common benign ovarian mass during pregnancy was the benign cystic teratoma (40%) [11]. Other common masses included corpus luteum and simple cysts (29%) [31]. Leiserowitz et al. [31] reported a malignancy rate of approximately 2%‐3% in ovarian masses during pregnancy. The predominant pathological types included epithelial ovarian cancer and undifferentiated tumors. A prospective study also found a malignancy rate of around 2% in ovarian masses during pregnancy, with epithelial ovarian cancer being the most common malignancy [7]. Our findings revealed that the most common benign ovarian tumor was the mature cystic teratoma, accounting for approximately 40%. The malignancy rate was 2.5%, primarily attributed to serous ovarian cancer. These results align with most relevant publications [7, 11].

In this study, ultrasound findings in the follow‐up group were mostly benign, typically simple cystic lesions with fine echogenic spots. In contrast, the surgical‐during‐pregnancy group exhibited more complex patterns, including cystic‐solid and mixed echoes. The only malignant case presented as a cystic‐solid mass with an irregular hypoechoic area on a septum and increased blood flow. Studies by Moro et al. [32, 33] showed that borderline ovarian tumors (BOTs) usually appear as unilocular‐solid or multilocular‐solid masses with papillary projections on ultrasound, and their features may overlap with noninvasive low‐grade serous carcinoma. And multilocular cysts with 2–10 locules suggest benign cystadenomas, whereas those with more than 10 locules may indicate gastrointestinal‐type BOTs. Ludovisi et al. [34] described serous surface papillary BOTs as a rare variant presenting as irregular solid lesions on the ovarian surface. Transvaginal ultrasound can effectively differentiate between benign and malignant ovarian masses in premenopausal women, even when the lesions are small [35, 36]. Previous studies have shown that transvaginal ultrasound can distinguish between invasive and noninvasive tumors, with a sensitivity of up to 100% and a specificity of 80% for detecting invasive tumors [37]. The systemic immune‐inflammation index (SIRI) and systemic inflammation response (SIR) scoring systems outperform CA125 in differentiating tumor types and provide more reliable noninvasive preoperative assessment [38]. In pregnant patients with ovarian masses, ultrasound‐based stratified management is safe and has minimal impact on obstetric outcomes [17, 39]. Therefore, standardized preoperative ultrasound evaluation is essential for improving the diagnosis and management of BOTs, especially in young and pregnant women, and for preserving fertility.

In conclusion, this study was the first report to observe the dynamic changes in the size of ovarian masses across different gestational stages. Younger age and higher prepregnancy BMI were found to be risk factors for the enlargement of ovarian masses during pregnancy. Given these risk factors, pregnant women with ovarian masses should undergo regular monitoring and follow‐up based on the dynamic changes in mass size. Additionally, different management strategies for ovarian masses during pregnancy did not demonstrate a significant difference in outcomes for either the pregnant women with ovarian masses or the newborns, and individualized management was crucial for pregnant women with ovarian masses.

## Funding

This work was supported by funding from Zhejiang Key Laboratory of Traditional Chinese Medicine for Diagnosis and Treatment of Gynecological Cancers (2022‐11).

## Ethics Statement

This study was approved by the Research Ethics Committee of the participating hospitals.

## Conflicts of Interest

The authors declare no conflicts of interest.

## Supporting Information

Additional supporting information can be found online in the Supporting Information section.

## Supporting information


**Supporting Information** Table S1: The size of ovarian masses during different trimesters. Table S2: Pathological types of ovarian masses during pregnancy in the surgery‐during‐pregnancy group.

## Data Availability

The data that support the findings of this study are available from the corresponding authors upon reasonable request.
