# Supplementary material for: Dynamic Changes of Ovarian Masses During Pregnancy and the Effects on Pregnancy Outcomes
Source: Nurs Res Pract. 2026 Jun 1;2026:6401831. doi: 10.1155/nrp/6401831 (PMC13239189; doi:10.1155/nrp/6401831)
Supplement: Supplementary file 1 — Supporting Information Table S1: The size of ovarian masses during different trimesters. Table S2: Pathological types of ovarian masses during pregnancy in the surgery‐during‐pregnancy group. [file NRP-2026-6401831-s001.docx]

|  | Maximum diameter (mm) | Volume (cm^3^) |
| --- | --- | --- |
| First trimester | 35.00 (28.00, 49.00) | 12.58 (6.24, 32.27) |
| Second trimester | 32.00 (0.00, 46.50) | 9.43 (0.00, 28.58) |
| Third trimester | 29.00 (0.00, 45.50) | 5.63 (0.00, 25.25) |
| Puerperium | 0.00 (0.00, 35.00) | 0.00 (0.00, 13.12) |

Table S1: The size of ovarian masses during different trimesters

Data were given as median (IQR).

| Types of Masses | n (%) |
| --- | --- |
| Mature Cystic Teratoma | 16 (40.0) |
| Mucinous Cystadenoma | 6 (15.0) |
| Endometrioma | 2 (5.0) |
| Serous Cystadenoma | 3 (7.5) |
| Simple Cyst | 3 (7.5) |
| Corpus Luteum Cyst | 5 (12.5) |
| Borderline Tumor | 1 (2.5) |
| Mixed (Serous and Mucinous) Cystadenoma | 2 (5.0) |
| Ovarian Cancer | 1 (2.5) |
| Ovarian Follicular Cyst | 1 (2.5) |

Table S2: Pathological types of ovarian masses during pregnancy in the surgery-during-pregnancy group

Data were given as n (%).
